# Picking up the pieces: Lessons learned about optimal public health and acute-care hospital collaboration during pandemics

**DOI:** 10.1017/ash.2023.184

**Published:** 2023-07-12

**Authors:** Krystina L. Woods, Bernard C. Camins

**Affiliations:** 1 Department of Infection Prevention, Mount Sinai West, New York, New York; 2 Division of Infectious Diseases, Department of Medicine, at Icahn School of Medicine, Mount Sinai, New York, New York; 3 Department of Infection Prevention, Mount Sinai Health System, New York, New York

Just before the coronavirus disease 2019 (COVID-19) pandemic, we marked the centenary of the 1918 Spanish Flu pandemic.^1^ We were preparing for outbreaks of highly infectious diseases, such as Ebola virus disease, with drills and protocol revisions. Before that, most healthcare epidemiologists and infection preventionists had close relationships with local departments of health (DOH) and public health agencies (PHAs). We reported specific infections to the DOH and orchestrated safe discharge plans for patients with communicable diseases such as tuberculosis (TB).

In 1893, New York City (NYC) became the first city in the United States to attempt to control the spread of TB through a combination of screening, reporting, treatment, and follow-up, including detention and court-ordered treatment of nonadherent individuals. By these means, NYC successfully decreased cases of TB. This effort was accomplished by adequate funding in addition to robust collaboration between physicians, hospitals, and the DOH. Similar efforts occurred nationally, and after a resurgence of TB in the 1990s, strategies for its control were laid out in a national plan.^2^

We became accustomed to PHAs providing timely, evidence-based, practical guidance, and developed positive working relationships with local DOH. Although the arrival of a respiratory viral pandemic was anticipated by experts, not until the COVID-19 pandemic did we realize that our preparedness and communication were suboptimal. This pandemic demonstrated the importance of succinct, truthful, and unified communication, not only for the consumption of the healthcare community but also for the public.

Given rapidly evolving information, at times we found ourselves hampered by PHAs guidance that in prior situations provided a significant amount of support and resources. However, working together through remote and recent pandemics created a path forward for more effective collaboration. Herein, we present lessons learned from recent intensive PHA collaboration by our large academic health system in NYC, during the COVID-19 and mpox emergencies.

## Gather feedback and align priorities

As with any successful undertaking, it is important to begin with an outline of goals and priorities, and a plan to achieve them. This makes expectations clear, serves to identify areas that require improvement or support, and holds all parties accountable to established outcomes. The process of setting those goals should ideally be a bidirectional conversation between the PHAs, local healthcare epidemiologists, and healthcare institutions. This communication would help to ensure that goals can be achieved and reflect everyone’s interests.

## Deliver clear and cohesive information to the general public and healthcare personnel

Once goals and priorities are defined, it is important to continue the 2-way dialogue to ensure that a workable plan exists to execute shared goals and to communicate them to the public. Early in the pandemic, we learned that public messaging needs to be clear, succinct, and coordinated so that it is not perceived as contradictory. It is equally important for PHAs to provide information to healthcare institutions that reflects the most current, realistic information, not an underestimate of the current state.

For example, when implementing mass vaccinations, as during COVID-19 or mpox, timely and transparent information from PHAs regarding the process for obtaining vaccines, who will administer the vaccine, and a mechanism to identify and vaccinate the highest risk groups would enable a less chaotic process. During the mpox outbreak in the summer of 2022, there was a large public interest in vaccination and providers referring patients for vaccination. However, clear and concise information from PHAs about the quantity of vaccines available or realistic estimates of when the limited supply might improve was not readily presented.^3^ Similarly, as COVID-19 vaccines were being distributed to healthcare-institutions as “vaccine points of delivery,” incomplete information was provided regarding the quantity a healthcare institution would receive, making scheduling patients’ vaccine appointments in advance almost impossible. At times, appointments were canceled, attracting the ire of the expectant public.^4^

When policy changes are made, it would be best for PHAs to consider how these changes will affect public perception and the healthcare-institutions’ ability to meet new requirements and at the same time keep patients safe during staffing shortages. For example, NYC implemented mitigation strategies like universal masking, social distancing, temperature and symptom checks, and later, vaccination requirements. Proof of vaccination or a recent test was required for indoor dining and entry into social and cultural events and spaces.^5^ COVID-19 vaccination and symptom and temperature screening requirements in New York have since been dropped in community settings. Retracting them in healthcare settings took considerably longer, stoking confusion, especially since it was well reported, even in the mainstream media, that temperature checks were ineffective.^6^ Similarly, despite the lack of evidence that routine screening tests for asymptomatic individuals prior to elective ambulatory surgical procedures are of benefit, they were required in New York State,^7^ and remained so for a considerable amount of time. Astute colleagues pointed out that relying on rapid antigen tests for preprocedure testing was ineffective given their poor sensitivity and rates of false-positive results, compounding the confusion.^8^

Consultation with healthcare-institutions would have been helpful in this situation because they had experience and knowledge of how to best apply testing in a variety of settings (e.g., before admission and before a procedure). We observed first-hand how these requirements posed a significant burden on patients, particularly those with limited access to testing, and led to delays in care, in addition to financial and staffing burdens to institutions.^9^ Improved dialogue between healthcare institutions and PHAs could highlight the challenges faced by healthcare institutions when presented with contradictory regulatory requirements that also send mixed messages to the public.

Policies that do not reflect current best practice, as they evolve quickly during a pandemic, frustrate practitioners and add to the perception that the medical establishment cannot be trusted. This discrepancy and lack of effective communication with the public have led to some healthcare workers being assaulted by visitors.^10^ Public health emergencies require buy-in from the public, making communication about changes crucial to get right at the outset. In an era of electronic information sharing and multiple modalities for communication, there should be a critical look at whether PHAs and healthcare institutions are leveraging all the tools available (e.g., community leaders and social media) in their communication with the public and with each other.

## Local hospital associations as intermediaries

The ultimate goals of PHAs and healthcare institutions are similar, although the path to achieving those goals may sometimes differ. Some PHA regulatory requirements cannot be achieved in the manner required; thus, seeking feedback to find a suitable compromise is essential. Although reasons may vary as to why a healthcare institution may not be able to meet the requirement completely, often it stems from a disconnect between the requirement and the daily workflow of a healthcare institution. Invariably, in the setting of a pandemic, healthcare institutions may be forced to pull resources from other endeavors to meet regulations that are incongruous with the workflow.

In the New York Metropolitan area, we were fortunate that the Greater New York Hospital Association (GNYHA) regularly convened a forum for healthcare epidemiologists from member hospitals to discuss topics of interest and concern even before the COVID-19 pandemic. This forum gave the healthcare epidemiologists responsible for implementing regulatory requirements an opportunity to discuss policies and share best practices as a counterpoint to the informal conversations that occurred in smaller groups. When COVID-19 and mpox arrived, this forum continued to serve as a resource for sharing ideas or solutions to collective challenges and as a venue for mutual support during trying times. The GNYHA provided a mechanism by which individual suggestions could be aggregated—and sometimes more importantly, anonymously—and conveyed to PHAs and government officials.

## Re-evaluate regulations routinely

PHAs should be open to feedback about the recommendations or regulations they set. They should commit to a plan to critically evaluate, at regular intervals, those policies to ensure that they reflect the current needs of healthcare institutions and the communities they serve, based on real-time clinical information. During a pandemic, needs shift quickly, often resulting in a situation where a regulatory requirement places an undue burden on healthcare institutions, rather than drive the change it was intended to accomplish. In the nascent days of the epidemic, testing for SARS-CoV-2 was limited and required approval from the PHA. Approval was only granted in cases of high pretest probability.^11^ Through dialogue with the PHA, the approval process was adjusted, to the benefit of all involved. When mpox began to spread rapidly, testing patients was significantly easier, partially because of lessons learned from COVID-19. However, treating mpox patients was arduous, given the volume of continuous documentation required.^12^ Although not all feedback from healthcare institutions needs to be implemented, some suggestions may be beneficial for all involved.

Ultimately, viewing the PHA and healthcare institution relationship as one based on collaboration and partnership, and strongly rooted in transparent, timely, data-driven, bidirectional dialogue, will lead to improvement in the care we provide our patients and communities. Cohesive messaging, especially during public health emergencies, from all those involved in healthcare will serve to engender the trust of the general public, whose cooperation is essential in a public health emergency (Table 1).

**Table 1.** Summary of Recommendations

Recommendation	Example(s) of An Ideal Collaborative State
Gather feedback and align priorities	PHAs and HIs convene to discuss planned recommendations and regulations and agree on deliverable metrics and implementation. PHAs should have a forum or venue for soliciting information from HIs, so that recommendations can be updated as data evolve.
Deliver clear and cohesive information to general public and healthcare personnel	PHAs make unified vaccination, testing or treatment plans available to the public, as early as possible. If recommendations for healthcare facilities are different from recommendations for the general public, PHAs should clearly state that as part of any announcement(s).
Re-evaluate regulations routinely	Meetings between PHAs and HIs should be set at regular, predetermined intervals during which information from the field can be presented to help modify recommendations and regulations. Meeting intervals should be adjusted to the pace and evolution of the current crisis—more frequent initially, becoming less frequent when appropriate.
Use local hospital associations as intermediaries	Seek feedback from local hospital associations because they can aggregate suggestions, comments, and observations from HEs and/or HIs anonymously.

Note. PHA, public health agency; HI, healthcare insitution; HE, healthcare epidemiologist.
